# 18F-FDG, 11C-Methionine, and 68Ga-Pentixafor PET/CT in Patients with Smoldering Multiple Myeloma: Imaging Pattern and Clinical Features

**DOI:** 10.3390/cancers12082333

**Published:** 2020-08-18

**Authors:** Xiang Zhou, Alexander Dierks, Olivia Kertels, Malte Kircher, Andreas Schirbel, Samuel Samnick, Andreas K. Buck, Sebastian Knorz, David Böckle, Lukas Scheller, Janin Messerschmidt, Mohammad Barakat, K. Martin Kortüm, Leo Rasche, Hermann Einsele, Constantin Lapa

**Affiliations:** 1 Department of Internal Medicine II, University Hospital of Würzburg, 97080 Würzburg, Germany; Zhou_X@ukw.de (X.Z.); knorz_s@ukw.de (S.K.); boeckle_d@ukw.de (D.B.); scheller_l@ukw.de (L.S.); Messerschm_j@ukw.de (J.M.); barakat_m@ukw.de (M.B.); Kortuem_M@ukw.de (K.M.K.); rasche_l@ukw.de (L.R.); Einsele_H@ukw.de (H.E.); 2 Department of Nuclear Medicine, University Hospital of Würzburg, 97080 Würzburg, Germany; dierks_a@ukw.de (A.D.); malte.kircher@uk-augsburg.de (M.K.); Schirbel_a@ukw.de (A.S.); samnick_S@ukw.de (S.S.); buck_a@ukw.de (A.K.B.); 3 Nuclear Medicine, Medical Faculty, University of Augsburg, 86156 Augsburg, Germany; 4 Department of Diagnostic and Interventional Radiology, University Hospital of Würzburg, 97080 Würzburg, Germany; kertels_o@ukw.de

**Keywords:** 18F-FDG PET/CT, 11C-Methionine PET/CT, 68Ga-Pentixafor PET/CT, smoldering myeloma

## Abstract

This study aimed to explore the correlation between imaging patterns and clinical features in patients with smoldering multiple myeloma (SMM) who simultaneously underwent 18F-FDG, 11C-Methionine, and 68Ga-Pentixafor positron emission tomography/computed tomography (PET/CT). We retrieved and analyzed clinical characteristics and PET imaging data of 10 patients with SMM. We found a significant correlation between bone marrow (BM) plasma cell (PC) infiltration and mean standardized uptake values (SUV_mean_) of lumbar vertebrae L2-L4 on 11C-Methionine PET/CT scans (*r* = 0.676, *p* = 0.031) and 68Ga-Pentixafor PET/CT scans (*r* = 0.839, *p* = 0.002). However, there was no significant correlation between BM involvement and SUV_mean_ of lumbar vertebrae L2-L4 on 18F-FDG PET/CT scans (*r* = 0.558, *p* = 0.093). Similarly, mean target-to-background ratios (TBR_mean_) of lumbar vertebrae L2-L4 also correlated with bone marrow plasma cell (BMPC) infiltration in 11C-Methionine PET/CT (*r* = 0.789, *p* = 0.007) and 68Ga-Pentixafor PET/CT (*r* = 0.724, *p* = 0.018) PET/CT. In contrast, we did not observe a significant correlation between BMPC infiltration rate and TBR_mean_ in 18F-FDG PET/CT (*r* = 0.355, *p* = 0.313). Additionally, on 11C-Methionine PET/CT scans, we found a significant correlation between BMPC infiltration and TBR_max_ of lumbar vertebrae L2-L4 (*r* = 0.642, *p* = 0.045). In conclusion, 11C-Methionine and 68Ga-Pentixafor PET/CT demonstrate higher sensitivity than 18F-FDG PET/CT in detecting BM involvement in SMM.

## 1. Introduction

Smoldering multiple myeloma (SMM) is an asymptomatic clonal plasma cell (PC) proliferative disorder, which is characterized by presence of monoclonal protein in serum and/or bone marrow (BM) PC infiltration of 10% to 60%, but in comparison to multiple myeloma (MM) without CRAB features (hypercalcemia, renal failure, anemia, and bone lesions) or myeloma-defining events (with bone marrow plasma cell (BMPC) infiltration ≥ 60%, serum involved/uninvolved free light chain (FLC) ratio ≥ 100, and ≥ 1 focal lesion, which is larger than 5 mm in size on Magnetic Resonance Imaging (MRI)) [1,2]. Other than monoclonal gammopathy of undetermined significance (MGUS), which also causes no symptoms, SMM inherits a higher risk of progression to symptomatic MM that requires treatment [3]. In a study of Kyle et al., more than 70% of the patients with SMM progressed to MM after a follow-up period of 15 years [4]. It has been reported in a variety of risk-stratification models that high BMPC infiltration rate is associated with shorter time to progression (TTP) in SMM patients [4,5,6]. These findings highlight the prognostic role of BMPC infiltration rate in SMM.

The current International Myeloma Working Group (IMWG) guidelines recommend whole-body computer tomography (CT) as the primary imaging modality to screen for osteolytic bone lesions in SMM and, if the whole-body CT is negative, whole-body MRI should be performed to exclude focal lesions as myeloma-defining events [7]. Apart from that, Positron Emission Tomography (PET)/CT represents an alternative method in place of CT or MRI to detect tumor metabolic activity. At present, 18F-fluorodeoxyglucose (18F-FDG) is the most widely used PET tracer in plasma cell disorders [7]. In SMM, Siontis et al. reported that patients who had a positive FDG PET/CT showed a 2-year progression rate of 75%, which was much higher than that in patients with a negative scan (30%) [8]. More recently, 11C-Methionine PET/CT and chemokine (C-X-C motif) receptor 4 (CXCR4) directed 68Ga-Pentixafor PET/CT have also been evaluated in symptomatic multiple myeloma (MM), and showed promising results for detection of intra- and extramedullary MM lesions when compared with standard 18F-FDG PET/CT [9,10,11,12]. However, the role of 11C-Methionine or 68Ga-Pentixafor PET/CT in SMM has not yet been explored.

Therefore, we performed a retrospective analysis of SMM patients who simultaneously underwent 18F-FDG, 11C-Methionine, and 68Ga-Pentixafor PET/CT. The aim of the current pilot study was to explore the potential correlation between imaging patterns and clinical features in SMM.

## 2. Methods

### 2.1. Patient Population

We collected data of patients suffering from SMM who underwent “triple-tracer” PET/CT (18F-FDG, 11C-Methionine, and 68Ga-Pentixafor) for diagnostic work-up at our institution between November 2015 and September 2016. SMM was diagnosed according to the current IMWG guidelines [1]. At the time point of imaging, BM biopsy from the posterior iliac crest was performed in all patients that were included into the analysis. The following data were collected and analyzed: patients’ demographic characteristics, time point of diagnosis, subtype of SMM, BMPC infiltration rate, cytogenetics, laboratory tests, and imaging data of PET/CT. High-risk cytogenetics was defined as per revised international staging system for MM, i.e., del(17p), t(4;14) and t(14;16) [13]. If the patients progressed to active MM in the course of the disease, data of treatment for MM were also analyzed. As per current guidelines, appearance of CRAB features and/or MM defining events was considered as progression to MM [1,2]. The clinical follow up data were evaluated as of May 2020. All procedures were performed under the conditions of the German pharmaceutical law (German Medicinal Products Act, AMG §13 2b) and in accordance with the responsible regulatory body (Regierung von Unterfranken, Germany) as well as in accordance with the current version of the Declaration of Helsinki as revised in 2013. All patients gave written informed consent prior to imaging procedures. Given the retrospective nature of this pilot analysis, the local ethics committee of University of Würzburg waived the need for additional approval.

### 2.2. Imaging Acquisition and Analysis

18F-FDG, 11C-MET, and 68Ga-Pentixafor were synthesized in-house as previously described [11,14]. PET/CT was performed on a PET/CT scanner (Siemens Biograph mCT 64, Siemens, Knoxville, TN, USA) within a median interval of two (range 0–26) days.

Patients fasted at least 4 hours before 18F-FDG (3 to 5 MBq/kg) and 11C-MET injection (6–10 MBq/kg). Prior to imaging with 68Ga-Pentixafor (2–3 MBq/kg), no fasting was required. PET/CT scans were acquired 60 min (18F-FDG, 68Ga-Petixafor) or 20 min (11C-MET) after tracer injection, using contrast-enhanced CT with dose modulation and a quality reference of 210 mAs or non-contrast-enhanced CT with Care Dose 4D and a quality reference of 80–120 mAs, including the skull to the proximal thighs and lower limbs. Consecutively, PET data were acquired in 3D-mode with 2 min emission time per bed position. After decay and scatter correction, PET data were reconstructed according to standard protocols consisting of 3D ordinary Poisson ordered-subset expectation maximization (OSEM) iterative reconstruction with time-of-flight and point spread function modeling, 3 iterations, 21 subsets, a 2 mm full-width at half-maximum Gaussian post-filter and a 200 × 200 image matrix.

First, a visual inspection of scans for elevated medullary tracer uptake was performed. BM involvement was defined as recently described [12,14]. In short, criteria to define a scan as PET-positive by FDG were those previously proposed by Nanni et al. [15], while for 11C-methionine and 68Ga-Pentixafor, BM involvement was visually determined as significantly increased tracer retention in the hematopoietic BM with or without the expansion of BM into distal parts of long bones [10,11,16]. An example of (near-)physiological tracer uptake in a patient (No. 7 in Table 1 and Table 2) with minimal malignant plasma cell infiltration (10%) is given in Figure S1.

For semi-quantitative analysis, standardized uptake values for mean medullary uptake (SUV_mean_) were determined as follows: In an initial step, transaxial slices in the middle of the lumbar vertebral bodies of L2-L4 were selected. Next, tracer uptake in each vertebral body was determined by placing a region-of-interest (ROI) of 10 mm diameter in the center of each vertebra. SUV_max_ and SUV_mean_ were calculated as the mean of the respective SUV_max_ and SUV_mean_ of these three ROI. 

For calculation of target-to-background ratios (TBR), a ROI of 15 mm diameter was placed in the center of the right atrium. Mean or maximum TBR (TBR_mean_ or TBR_max_) were calculated by dividing the SUV_mean_ or SUV_max_ of the lumbar vertebral bodies L2-L4 by the SUV_mean_ of the right atrium.

### 2.3. Statistical Analysis

Patients’ characteristics were summarized as absolute number and percentage or, if not otherwise stated, as median and range. We used a linear regression model to evaluate the correlation between BMPC infiltration rates and mean standardized uptake value (SUV_mean_) or mean target to background ratio (TBR_mean_) in these patients. Pearson’s correlation coefficient (Pearson’s *r*) was calculated. The Kaplan–Meier method was used to analyze the survival outcome of the patients. Time to progression (TTP) was defined as the time period between PET/CT scans and the time point of progression to MM or the last follow up. A two-tailed log-rank test was used to compare the survival outcomes between the subgroups. These analyses were performed with GraphPad Prism 5.0. A *p*-value of <0.05 was considered statistically significant.

## 3. Results

### 3.1. Patients’ Characteristics

In total, we identified ten patients with SMM to be included in our retrospective analysis. Patients’ characteristics and treatment are summarized in Table 1. The median age at diagnosis of SMM was 62 (range, 41–74) years, and 80% of the patients (*n* = 8) were male. Six (60%), two (20%), and two (20%) patients had IgG, IgA, and LC subtype of SMM, respectively. At the time point of imaging, BM biopsy revealed a median BMPC infiltration rate of 16% (range: 10–40%) in our patients. Only one (10%) patient presented a relatively high rate of BMPC infiltration of 40%, while the BMPC infiltration rates were ≤20% in the other nine (90%) patients. Cytogenetic data were available in six patients. Two (20%) patients showed high-risk cytogenetics t(4;14), and the other four (40%) patients had standard-risk cytogenetics. The median of follow up time was 46 months (range: 9–51 months). Overall, three (30%) patients progressed to MM. Among these three patients, two (20%) met the criteria serum involved/uninvolved free light chain (FLC) ratio ≥ 100, and pathologic fracture was present in one (10%) patient. On the patient’s request, one (10%) patient did not receive any treatment. The other two patients were treated with VCD (bortezomib, cyclophosphamide, and dexamethasone) and PAD-Rev (bortezomib, doxorubicin, lenalidomide, and dexamethasone), respectively. Following induction therapy, both patients underwent high-dose melphalan and autologous stem cell transplant. Notably, in the course of the disease, the both patients harboring t(4;14) and consequently high-risk cytogenetics developed MM that required treatment according to the current IMWG recommendation [1].

### 3.2. Imaging Patterns and Clinical Features

Imaging parameters and results from 18F-FDG, 11C-Methionine, and 68Ga-Pentixafor PET/CT scans are summarized in Table 2. Overall, all patients displayed physiological tracer uptake on 18F-FDG PET/CT scans so that they were classified as negative for MM lesions. However, two (20%) and five (50%) patients showed increased tracer uptake in the entire skeleton on 11C-Methionine, and 68Ga-Pentixafor PET/CT scans, respectively. We did not observe any difference in TTP between “triple-negative” patients, i.e., 18F-FDG, 11C-Methionine, and 68Ga-Pentixafor PET/CT scans, and the others (*p* = 0.91).

**Figure 1:**
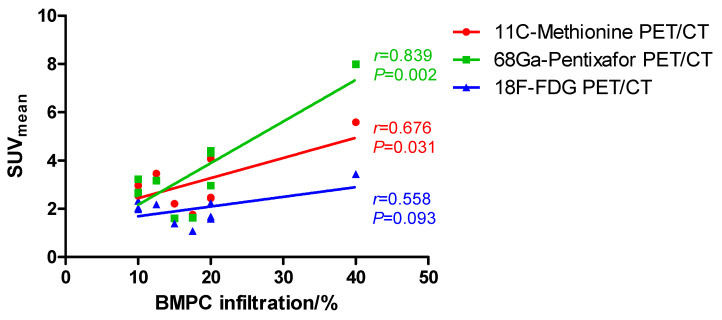
Correlation between bone marrow plasma cell (BMPC) infiltration and mean standardized uptake value (SUV_mean_) of lumbar vertebrae (LV) L2-L4 on 11C-Methionine, 68Ga-Pentixafor, and 18F-FDG PET/CT: A significant correlation between BMPC infiltration and SUV_mean_ of LV L2-L4 was shown by 11C-Methionine PET/CT (Pearson’s *r* = 0.676, *p* = 0.031, red) and 68Ga-Pentixafor (Pearson’s *r* = 0.839, *p* = 0.002, green) PET/CT. In contrast, there was no significant correlation between BMPC infiltration and SUV_mean_ of LV L2-L4 on 18F-FDG PET/CT (Pearson’s *r* = 0.558, *p* = 0.093, blue).

**Figure 2:**
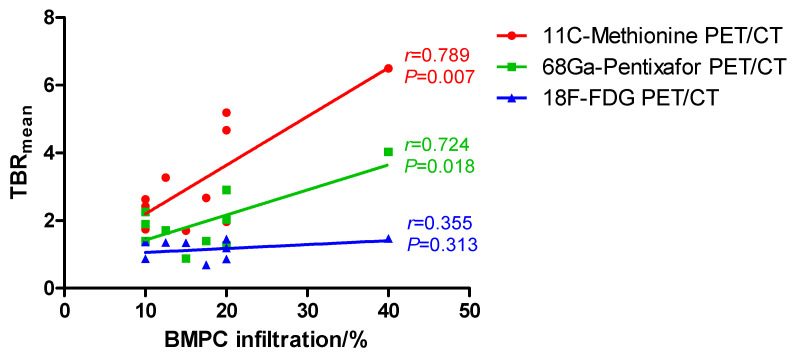
Correlation between bone marrow plasma cell (BMPC) infiltration and mean target to background ratio (TBR_mean_) on 11C-Methionine, 68Ga-Pentixafor, and 18F-FDG PET/CT: We observed a significant correlation between BMPC infiltration and TBR_mean_ in 11C-Methionine PET/CT (Pearson’s *r* = 0.789, *p* = 0.007, red) and 68Ga-Pentixafor (Pearson’s *r* = 0.724, *p* = 0.018, green) PET/CT. However, BMPC infiltration did not correlate with TBR_mean_ in 18F-FDG PET/CT (Pearson’s *r* = 0.355, *p* = 0.313, blue).

We further explored the correlation between BMPC infiltration rates and SUV_mean_ or TBR_mean_ of lumbar vertebrae L2-L4 on 11C-Methionine, 68Ga-Pentixafor, and 18F-FDG PET/CT scans. We found a significant correlation between BMPC infiltration and SUV_mean_ of lumbar vertebrae L2-L4 on 11C-Methionine PET/CT scans (Pearson’s *r* = 0.676, *p* = 0.031, red line in Figure 1) and 68Ga-Pentixafor PET/CT scans (Pearson’s *r* = 0.839, *p* = 0.002, green line in Figure 1). However, there was no significant correlation between BM involvement and SUV_mean_ of lumbar vertebrae L2-L4 on 18F-FDG PET/CT scans (Pearson’s *r* = 0.558, *p* = 0.093, blue line in Figure 1). Similarly, TBR_mean_ of lumbar vertebrae L2-L4 also correlated with BMPC infiltration rate in 11C-Methionine PET/CT (Pearson’s *r* = 0.789, *p* = 0.007, red line in Figure 2) and 68Ga-Pentixafor PET/CT (Pearson’s *r* = 0.724, *p* = 0.018, green line in Figure 2) PET/CT, but not in 18F-FDG PET/CT (Pearson’s *r* = 0.355, *p* = 0.313, blue line in Figure 2).

**Figure 3:**
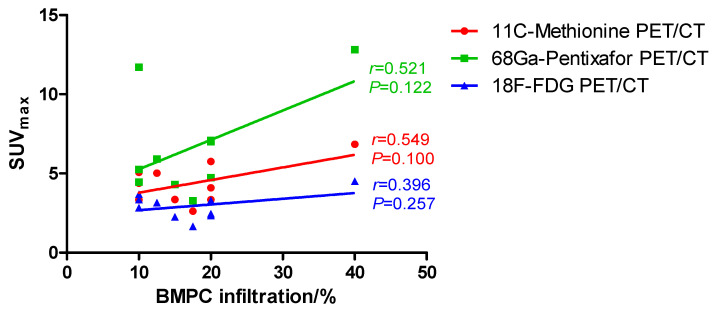
Correlation between bone marrow plasma cell (BMPC) infiltration and maximum standardized uptake value (SUV_max_) of lumbar vertebrae (LV) L2-L4 on 11C-Methionine, 68Ga-Pentixafor, and 18F-FDG PET/CT: We did not find a significant correlation between BMPC infiltration and SUV_max_ of LV L2-L4 on 11C-Methionine PET/CT (Pearson’s *r* = 0.549, *p* = 0.100, red), 68Ga-Pentixafor (Pearson’s *r* = 0.521, *p* = 0.122, green), and 18F-FDG PET/CT (Pearson’s *r* = 0.396, *p* = 0.257, blue).

**Figure 4:**
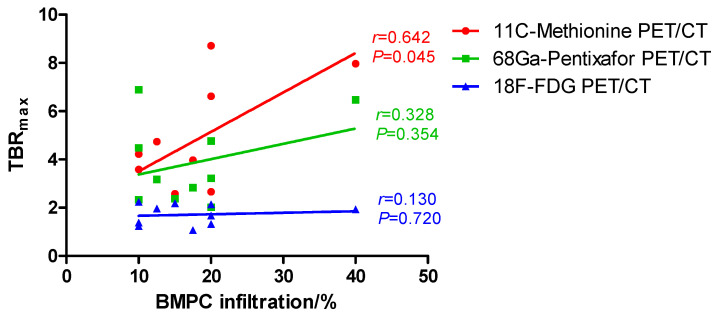
Correlation between bone marrow plasma cell (BMPC) infiltration and maximum target to background ratio (TBR_max_) on 11C-Methionine, 68Ga-Pentixafor, and 18F-FDG PET/CT: We observed a significant correlation between BMPC infiltration and TBR_max_ in 11C-Methionine PET/CT (Pearson’s *r* = 0.642, *p* = 0.045, red). On 68Ga-Pentixafor (Pearson’s *r* = 0.328, *p* = 0.354, green) and 18F-FDG (Pearson’s *r* = 0.130, *p* = 0.720, blue) PET/CT, the correlation between TBR_max_ and BMPC infiltration was not significant.

The correlation between BMPC infiltration and SUV_max_ or TBR_max_ on 11C-Methionine, 68Ga-Pentixafor, and 18F-FDG PET/CT scans was also analyzed. On 11C-Methionine PET/CT scans, there was a tendency toward a correlation between SUV_max_ of lumbar vertebrae L2-L4 and BMPC infiltration (Pearson’s *r* = 0.549, *p* = 0.100, red line in Figure 3) and, moreover, we found a significant correlation between BMPC infiltration rate and TBR_max_ of lumbar vertebrae L2-L4 (Pearson’s *r* = 0.642, *p* = 0.045, red line in Figure 4). In contrast, the correlation between BM involvement and SUV_max_ or TBR_max_ of lumbar vertebrae L2-L4 on 68Ga-Pentixafor (SUV_max_: Pearson’s *r* = 0.521, *p* = 0.122, green line in Figure 3; TBR_max_: Pearson’s *r* = 0.328, *p* = 0.354, green line in Figure 4) and 18F-FDG PET/CT scans (SUV_max_: Pearson’s *r* = 0.396, *p* = 0.257, blue line in Figure 3; TBR_max_: Pearson’s *r* = 0.130, *p* = 0.720, blue line in Figure 4) was not significant.

**Figure 5:**
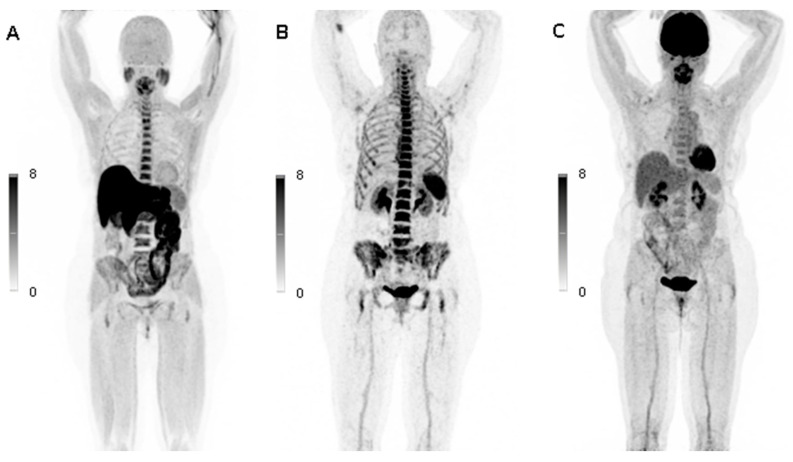
11C-Methionine, 68Ga-Pentixafor, and 18F-FDG PET/CT scans in a patient with smoldering multiple myeloma (SMM): The figure shows a patient (No. 1 in Table 1 and Table 2) with SMM who had a bone marrow plasma cell infiltration rate of 40%. 11C-Methionine (**A**) and 68Ga-Pentixafor PET/CT scans (**B**) presented increased tracer uptake which correlated with plasma cell infiltration (e.g., spine and pelvis), while the standard 18F-FDG PET/CT scan (**C**) showed physiological tracer uptake in the entire skeleton.

In our cohort, there were three patients who developed MM in the course of the disease (patient No. 1, 3, and 9). In patient No. 1, who had a BMPC infiltration rate of 40%, both 11C-Methionine and 68Ga-Pentixafor PET/CT scans demonstrated an increased tracer uptake (e.g., spine and pelvis), while standard 18F-FDG PET/CT remained negative showing physiological FDG uptake (Figure 5). Interestingly, all three 18F-FDG, 11C-Methionine, and 68Ga-Pentixafor PET/CT scans were negative in patient No. 3 who exhibited t(4;14) and developed MM with pathologic fracture of lumbar vertebra L3 13 months later. In the patient No. 9, the serum involved/uninvolved FLC ratio increased to ≥100 after a follow up of 14 months.

## 4. Discussion

We analyzed the data of patients with SMM, in whom 18F-FDG, 11C-Methionine, and 68Ga-Pentixafor PET/CT were performed, with the aim to explore the relationship between imaging patterns and clinical features in SMM. To the best of our knowledge, this is the first study evaluating this “triple-tracer” PET imaging in patients with SMM.

In our cohort, both patients with t(4;14) progressed to MM after a follow up of 13 and 14 months, respectively. It has been reported that the proportion of clonal PC harboring t(4;14) increases with progression from MGUS or SMM to active MM and, therefore, t(4;14) may be a potential driver of disease progression in patients with SMM [17]. In addition, Neben et al. found that t(4;14) was an adverse prognostic factor indicating an significantly inferior TTP in patients with SMM [18]. Our finding is in line with these previous publications.

Overall, BMPC infiltration rate positively correlated with SUV_mean_ and TBR_mean_ of lumbar vertebrae L2-L4 on CXCR4 directed 68Ga-Pentixafor and 11C-Methionine PET/CT scans, and we also observed a significant correlation between BM involvement and TBR_max_ on 11C-Methionine PET/CT scans. However, this correlation was not significant in standard 18F-FDG PET/CT. CXCR4 is a molecule expressed on PC, which plays a crucial role in the interaction between MM cells and BM microenvironment [19]. In MM, CXCR4 overexpression is associated with disease progression and adverse outcome [20,21]. Comparably, in active MM, a study of Pan et al. suggested a positive correlation between tumor burden and SUV_mean_ on 68Ga-Pentixafor PET/CT scans, and 68Ga-Pentixafor PET/CT showed higher sensitivity than standard 18F-FDG PET/CT in detecting focal lesions or diffuse BM involvement [12]. Similarly, Lapa et al. reported that SUV_mean_ on both 11C-Methionine and 18F-FDG PET/CT scans correlated with BMPC infiltration rate, with 11C-Methionine PET/CT demonstrating a stronger correlation and therefore a higher sensitivity [9,10]. Currently, posterior iliac crest remains the standard site for BM biopsy, and the axial skeleton, including the spine and pelvis, are the most common sites of BMPC infiltration in MM, suggesting that SUV and TBR of lumbar vertebrae might also be representative for BM involvement confirmed by conventional BM biopsy. Altogether, our results provide the first evidence of the higher sensitivity of 68Ga-Pentixafor and 11C-Methionine PET/CT in detecting BM involvement also in SMM. Importantly, other than BM biopsy, PET/CT is a non-invasive diagnostic method. However, due to the small sample size, the prognostic value of imaging positivity in 68Ga-Pentixafor or 11C-Methionine PET/CT could not be elucidated here. Further investigation of this “triple-tracer” approach is needed at this point to early identify high-risk SMM patients who represent potential candidates for clinical trials.

In our analysis, 18F-FDG PET/CT was negative in all patients. We observed a patient with high-risk cytogenetic marker and rapid progression to active MM, who showed negative results on all three PET scans. On the other hand, there were also patients with positive 11C-Methionine or 68Ga-Pentixafor PET, who had standard-risk cytogenetics and did not progress to MM. A possible explanation might be that the negative PET imaging could be “masked” by rapid disease progression of high-risk SMM as suggested by Neben et al. [18]. In brief, the sensitivity and specificity of 18F-FDG, 11C-Methionine, or 68Ga-Pentixafor PET should be further evaluated.

There are several limitations of our pilot study. First, the current study is based on a small number of patients. Since every single case of our cohort can significantly affect the statistical analysis all results have to be interpreted with extreme caution and the prognostic value of imaging cannot be evaluated. Second, image analysis was based on conventional parameters including SUV and TBR, and more sophisticated measures, such as total metabolic volumes or total lesion glycolysis, that might provide a more comprehensive estimation of tumor burden, were not explored in this pilot study. However, we believe that our pilot study might stimulate further research in order to non-invasively identify and characterize high-risk SMM patients using whole-body imaging.

## 5. Conclusions

In conclusion, 11C-Methionine and 68Ga-Pentixafor PET/CT demonstrate higher sensitivity than 18F-FDG PET/CT in detecting BM involvement also in SMM. 11C-Methionine and 68Ga-Pentixafor PET/CT might be an option for non-invasive BM infiltration assessment and might help to identify high BMPC infiltration rates in SMM. Further investigations of this “triple-trace” PET imaging in SMM are warranted.

## Figures and Tables

**Table 1:** Patients’ characteristics.

Case	Age at Diagnosis, Years	Sex	SMM Subtye	BMPC Infiltration Rate	Cytogenetics	Crea, mg/dL	Hb, g/dL	LDH, IU/L	B2M, mg/L	Calcium, mmol/L	Time of Follow Up in Months	Progression to MM	MM Defining Events	First Line Therapy
1	58	f	LC λ	40%	NA	0.65	12.9	200	2.0	2.5	51	Yes	λ/κ FLC ratio ≥100	None *
2	74	f	LC λ	10%	t (11;14)	0.77	11.6	219	1.7	2.3	50	No	None	None
3	65	m	IgG κ	15%	t (4;14), gain1q21	0.86	14.2	143	1.5	2.3	51	Yes	Bone lesion	VCD + ASCT
4	57	m	IgA κ	10%	HDMM, gain1q21	0.63	12.1	204	2.2	2.5	48	No	None	None
5	71	m	IgG κ	17.5%	NA	1.20	12.4	167	3.3	2.3	47	No	None	None
6	54	m	IgG κ	20%	NA	0.90	13.8	165	NA	2.3	44	No	None	None
7	44	m	IgA λ	10%	NA	0.89	15.6	154	1.7	2.5	37	No	None	None
8	66	m	IgG κ	12.5%	HDMM	1.10	13.2	156	2.0	2.4	9	No	None	None
9	41	m	IgG λ	20%	t (4;14), del13q14	0.99	15.5	155	1.8	2.5	43	Yes	λ/κ FLC ratio ≥100	PAD-Rev + ASCT
10	69	m	IgG λ	20%	t (11;14)	1.45	10.5	259	3.3	2.4	41	No	None	None

ASCT: autologous stem cell transplant; B2M-β2: microglobulin; BMPC: bone marrow plasma cell; Crea: creatinine; f: female; FLC: free light chain; Hb: hemoglobin, HDMM: hyperdiploid multiple myeloma; LC: light chain; LDH: lactate dehydrogenase; m: male; MM: multiple myeloma; NA: not available; PAD: Rev-bortezomib, doxorubicin, lenalidomide, dexamethasone; SMM: smoldering multiple myeloma; VCD: bortezomib, cyclophosphamide, dexamethasone; * Not treated on patient’s request.

**Table 2:** Imaging parameters.

Case	18F-FDG PET/CT						11C-Methionine PET/CT						68Ga-Pentixafor PET/CT					
	PET	SUV_max_ of LV L2-L4	SUV_mean_ of LV L2-L4	SUV_mean_ in RA	TBR_max_	TBR_mean_	PET	SUV_max_ of LV L2-L4	SUV_mean_ of LV L2-L4	SUV_mean_ in RA	TBR_max_	TBR_mean_	PET	SUV_max_ of LV L2-L4	SUV_mean_ of LV L2-L4	SUV_mean_ in RA	TBR_max_	TBR_mean_
1	neg	4.50	3.44	2.33	1.93	1.47	pos	6.85	5.59	0.86	7.97	6.50	pos	12.82	7.99	1.98	6.47	4.03
2	neg	2.83	1.98	2.28	1.24	0.87	neg	3.33	2.54	1.46	2.28	1.74	neg	4.46	2.67	1.91	2.33	1.40
3	neg	2.26	1.39	1.04	2.18	1.34	neg	3.36	2.21	1.30	2.58	1.70	neg	4.31	1.60	1.82	2.37	0.88
4	neg	3.68	2.33	2.66	1.38	0.87	neg	4.38	2.96	1.22	3.59	2.42	pos	11.71	3.23	1.70	6.89	1.90
5	neg	1.66	1.08	1.56	1.07	0.69	pos	2.62	1.76	0.66	3.97	2.67	neg	3.28	1.63	1.16	2.83	1.40
6	neg	2.35	1.68	1.41	1.67	1.19	neg	4.09	2.44	0.47	8.71	5.19	pos	6.99	4.40	2.17	3.22	2.03
7	neg	3.34	2.04	1.49	2.24	1.37	neg	5.07	3.16	1.20	4.22	2.63	neg	5.25	2.64	1.17	4.48	2.26
8	neg	3.16	2.18	1.61	1.96	1.35	neg	5.02	3.46	1.06	4.74	3.27	neg	5.90	3.17	1.86	3.17	1.71
9	neg	3.29	2.23	1.54	2.14	1.45	neg	5.76	4.07	0.87	6.62	4.67	pos	7.09	4.32	1.49	4.76	2.90
10	neg	2.45	1.59	1.85	1.32	0.86	neg	3.35	2.47	1.26	2.66	1.96	pos	4.73	2.96	2.34	2.02	1.27

FDG: flurodeoxyglucose; LV: lumbar vertebrae; neg: negative; PET/CT: Positron Emission Tomography/Computer Tomography; pos: positive; RA: right atrium; SUV_max_: maximum standardized uptake value; SUV_mean_: mean standardized uptake value; TBR_max_: maximum target to background ratio; TBR_mean_: mean target to background ratio.

## Supplementary Materials

The following are available online at https://www.mdpi.com/2072-6694/12/8/2333/s1, Figure S1: 11C-Methionine, 68Ga-Pentixafor, and 18F-FDG PET/CT scans in a patient 10 with smoldering multiple myeloma (SMM).

## References

[1] Rajkumar S.V., Dimopoulos M.A., Palumbo A., Blade J., Merlini G., Mateos M.V., Kumar S., Hillengass J., Kastritis E., Richardson P. (2014). International Myeloma Working Group updated criteria for the diagnosis of multiple myeloma. Lancet Oncol..

[2] Mateos M.V., Gonzalez-Calle V. (2017). Smoldering Multiple Myeloma: Who and When to Treat. Clin. Lymphoma Myeloma Leuk..

[3] Rajkumar S.V., Landgren O., Mateos M.V. (2015). Smoldering multiple myeloma. Blood.

[4] Kyle R.A., Remstein E.D., Therneau T.M., Dispenzieri A., Kurtin P.J., Hodnefield J.M., Larson D.R., Plevak M.F., Jelinek D.F., Fonseca R. (2007). Clinical course and prognosis of smoldering (asymptomatic) multiple myeloma. N. Engl. J. Med..

[5] Cocito F., Mangiacavalli S., Ferretti V.V., Cartia C.S., Ganzetti M., Benveuti P., Pompa A., Catalano M., Fugazza E., Landini B. (2019). Smoldering multiple myeloma: The role of different scoring systems in identifying high-risk patients in real-life practice. Leuk. Lymphoma.

[6] Lakshman A., Rajkumar S.V., Buadi F.K., Binder M., Gertz M.A., Lacy M.Q., Dispenzieri A., Dingli D., Fonder A.L., Hayman S.R. (2018). Risk stratification of smoldering multiple myeloma incorporating revised IMWG diagnostic criteria. Blood Cancer J..

[7] Hillengass J., Usmani S., Rajkumar S.V., Durie B.G.M., Mateos M.V., Lonial S., Joao C., Anderson K.C., Garcia-Sanz R., Riva E. (2019). International myeloma working group consensus recommendations on imaging in monoclonal plasma cell disorders. Lancet Oncol..

[8] Siontis B., Kumar S., Dispenzieri A., Drake M.T., Lacy M.Q., Buadi F., Dingli D., Kapoor P., Gonsalves W., Gertz M.A. (2015). Positron emission tomography-computed tomography in the diagnostic evaluation of smoldering multiple myeloma: Identification of patients needing therapy. Blood Cancer J..

[9] Lapa C., Garcia-Velloso M.J., Luckerath K., Samnick S., Schreder M., Otero P.R., Schmid J.S., Herrmann K., Knop S., Buck A.K. (2017). (11)C-Methionine-PET in Multiple Myeloma: A Combined Study from Two Different Institutions. Theranostics.

[10] Lapa C., Knop S., Schreder M., Rudelius M., Knott M., Jorg G., Samnick S., Herrmann K., Buck A.K., Einsele H. (2016). 11C-Methionine-PET in Multiple Myeloma: Correlation with Clinical Parameters and Bone Marrow Involvement. Theranostics.

[11] Lapa C., Schreder M., Schirbel A., Samnick S., Kortum K.M., Herrmann K., Kropf S., Einsele H., Buck A.K., Wester H.J. (2017). [(68)Ga]Pentixafor-PET/CT for imaging of chemokine receptor CXCR4 expression in multiple myeloma - Comparison to [(18)F]FDG and laboratory values. Theranostics.

[12] Pan Q., Cao X., Luo Y., Li J., Feng J., Li F. (2020). Chemokine receptor-4 targeted PET/CT with (68)Ga-Pentixafor in assessment of newly diagnosed multiple myeloma: Comparison to (18)F-FDG PET/CT. Eur. J. Nucl. Med. Mol. Imaging.

[13] Palumbo A., Avet-Loiseau H., Oliva S., Lokhorst H.M., Goldschmidt H., Rosinol L., Richardson P., Caltagirone S., Lahuerta J.J., Facon T. (2015). Revised International Staging System for Multiple Myeloma: A Report From International Myeloma Working Group. J. Clin. Oncol..

[14] Morales-Lozano M.I., Viering O., Samnick S., Rodriguez-Otero P., Buck A.K., Marcos-Jubilar M., Rasche L., Prieto E., Kortum K.M., San-Miguel J. (2020). (18)F-FDG and (11)C-Methionine PET/CT in Newly Diagnosed Multiple Myeloma Patients: Comparison of Volume-Based PET Biomarkers. Cancers.

[15] Nanni C., Versari A., Chauvie S., Bertone E., Bianchi A., Rensi M., Bello M., Gallamini A., Patriarca F., Gay F. (2018). Interpretation criteria for FDG PET/CT in multiple myeloma (IMPeTUs): Final results. IMPeTUs (Italian myeloma criteria for PET USe). Eur. J. Nucl. Med. Mol. Imaging.

[16] Dankerl A., Liebisch P., Glatting G., Friesen C., Blumstein N.M., Kocot D., Wendl C., Bunjes D., Reske S.N. (2007). Multiple Myeloma: Molecular Imaging with 11C-Methionine PET/CT--Initial Experience. Radiology.

[17] Lopez-Corral L., Gutierrez N.C., Vidriales M.B., Mateos M.V., Rasillo A., Garcia-Sanz R., Paiva B., San Miguel J.F. (2011). The progression from MGUS to smoldering myeloma and eventually to multiple myeloma involves a clonal expansion of genetically abnormal plasma cells. Clin. Cancer Res..

[18] Neben K., Jauch A., Hielscher T., Hillengass J., Lehners N., Seckinger A., Granzow M., Raab M.S., Ho A.D., Goldschmidt H. (2013). Progression in smoldering myeloma is independently determined by the chromosomal abnormalities del(17p), t(4;14), gain 1q, hyperdiploidy, and tumor load. J. Clin. Oncol..

[19] Peled A., Klein S., Beider K., Burger J.A., Abraham M. (2018). Role of CXCL12 and CXCR4 in the pathogenesis of hematological malignancies. Cytokine.

[20] Waldschmidt J.M., Simon A., Wider D., Muller S.J., Follo M., Ihorst G., Decker S., Lorenz J., Chatterjee M., Azab A.K. (2017). CXCL12 and CXCR7 are relevant targets to reverse cell adhesion-mediated drug resistance in multiple myeloma. Br. J. Haematol..

[21] Vande Broek I., Leleu X., Schots R., Facon T., Vanderkerken K., Van Camp B., Van Riet I. (2006). Clinical significance of chemokine receptor (CCR1, CCR2 and CXCR4) expression in human myeloma cells: The association with disease activity and survival. Haematologica.

