# Supplementary material for: 18F-FDG, 11C-Methionine, and 68Ga-Pentixafor PET/CT in Patients with Smoldering Multiple Myeloma: Imaging Pattern and Clinical Features
Source: Cancers (Basel). 2020 Aug 18;12(8):2333. doi: 10.3390/cancers12082333 (PMC7465161; doi:10.3390/cancers12082333)
Supplement: Supplementary file 1 [file cancers-12-02333-s001.pdf]

Supplementary Materials

18F-FDG, 11C-Methionine, and 68Ga-Pentixafor PET/CT in Patients with Smoldering Multiple Myeloma: Imaging Pattern and Clinical Features

Xiang Zhou, Alexander Dierks, Olivia Kertels, Malte Kircher, Andreas Schirbel, Samuel Samnick, Andreas K Buck, Sebastian Knorz, David Böckle, Lukas Scheller, Janin Messerschmidt, Mohammad Barakat, K Martin Kortüm, Leo Rasche, Hermann Einsele and Constantin Lapa

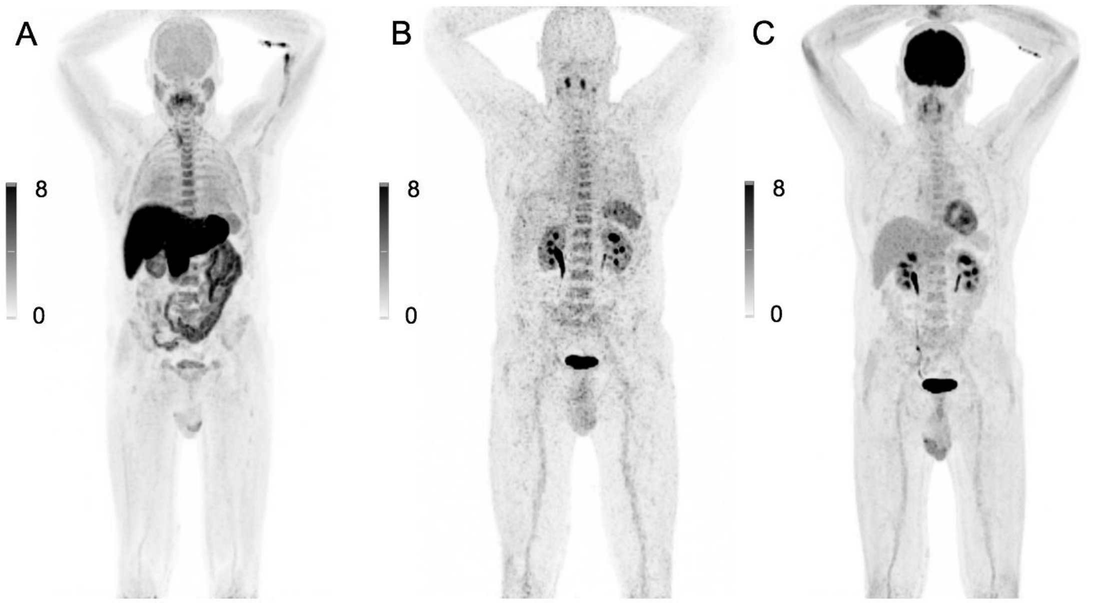

Figure S1. 11C-Methionine, 68Ga-Pentixafor, and 18F-FDG PET/CT scans in a patient with smoldering multiple myeloma (SMM). The figure shows a patient (No. 7 in Table 1) with SMM who had a bone marrow plasma cell infiltration rate of 10%. All three scans, i.e. 11C-Methionine (**A**), 68Ga-Pentixafor PET/CT (**B**) and 18F-FDG PET/CT (**C**) scans showed physiological tracer uptake in the entire skeleton.

© 2020 by the authors. Licensee MDPI, Basel, Switzerland. This article is an open access article distributed under the terms and conditions of the Creative Commons Attribution (CC BY) license (http://creativecommons.org/licenses/by/4.0/).
